# Mexico: Determinants of the real exchange rate, 2001.01–2022.12

**DOI:** 10.1371/journal.pone.0286331

**Published:** 2023-12-06

**Authors:** Eduardo Loría, Lorenzo Nalin

**Affiliations:** School of Economics, National Autonomous University of Mexico (UNAM), Mexico City, Mexico; Northeast Normal University, CHINA

## Abstract

We estimate the determinants (terms of trade, tradable to non-tradable price differentials, interest rate differentials, forward exchange rate and risk premium) of the Mexican bilateral real exchange rate (*q*) for the short and long run by using an Autoregressive Distributed Lag model (ARDL, Pesaran and Shin et al. (2001)) for Mexico (2001.01–2022.12). The inclusion of commercial and financial variables and finding empirical evidence of cointegration only for 2009.01–2022.12 are the main contributions. Our results indicate no cointegrating relationship either for the entire sample, or for 2001.01–2008.12. This finding has to do with the increasing international financialization process, after the 2008–2009 *Great Financial Crisis*. Using a double log model we find that: a) there is a strong short-run autoregressive effect of *q* of up to 4 lags (0.75), b) that the *Balassa-Samuelson Effect* is the largest in the model (-0.27 and -1.11 for short and long terms), c) the next most important factor is the terms of trade (-0.126 and -0.51, respectively), d) there are considerable, although lesser, effects of financial variables: forward exchange rate (0.0155 and 0.063, respectively) and risk-premium (0.009 and 0.036, respectively), e) there is a clear long-term trend of real depreciation expressed by the trend of 0.0020, which suggests that the PPP hypothesis applies.

## Introduction

**Once instability is understood as a theoretical possibility, then we are in a position to design appropriate interventions to constrain it**

**Hyman Minsky**

For the classical school of thought, the exchange rate is just another price determined by the foreign exchange market, which reflects trade with the outside world and its flexibility allows to absorb the shocks that every economy tends to face. Therefore, in this approach, it is essentially an endogenous variable and it does not act as an economic policy instrument. Carrière‐Swallow *et al*. [1], based on a sample of 196 countries for the period 1990–2016, provide strong evidence that the exchange rate plays a key role of a shock absorber by reducing the response of output to changes in the terms of trade to which emerging countries are more sensitive.

Conversely, for *development macroeconomics* the active management of the exchange rate has a strategic role in promoting technical progress, investment and growth (Rapetti *et al*. [2, 3], Razmi *et al*. [4], Rodrik [5], Bresser-Pereira [6], Capriata and Flauzino [7], Gala [8], Goda and Priewe [9]). However, it has also been advised on the potential pitfalls and volatility of the nominal exchange rate and, thus, inflationary pressures coming from sistematic depreciations, [5, 10].

Since the 1995 Mexican Tequila Crisis, the vast majority of developing countries have avoided huge *q* depreciations because of their high inflationary costs and the deterioration in the living standards of the poorest share of the population.

For the *New Developmentalism* theory, managing the depreciated *q* is an important industrial policy tool that accelerates growth of emerging countries; China and India, for example, have experienced very rapid growth over the past decades. This is because they consider that the real exchange rate is not just another price of the economic system, but rather a determining variable of industrial policy and of economic development because it fulfills the Marshall-Lerner condition and avoids the Dutch Disease. This line of theory sustains that exchange rate appreciations have long-term de-industrializing and impoverishing effects, because it is believed that, insofar as inflation in emerging countries (particularly in Latin America) is higher than in the developed world, growth processes lead to exchange rate appreciations, which reduce demand for domestic goods in favor of goods of foreign origin and increase the use of imported capital against employment. So, if the exchange rate is used as an "inflation anchor" rather than a tool for growth, exchange rate appreciations that will occur will be similar to large currency inflows that cause Dutch Disease-associated effects and significant reductions in growth, Bresser-Pereira [6].

Thus, in this approach, the active management of *q* has growth effects due to the fact that, in the long run, there are redistributive gains, because, although inflation caused by depreciations in the short run deteriorates distribution, growth effects that it should generate outweigh the concentrating effects, Bresser-Pereira [6].

Due to the financial integration, there is an increasing volatility in the nominal exchange rate (*E*) and in *q*, so we seek to explore its main commercial and financial short- and long-run determinants. We claim that both factors are increasingly important to determine its short-terms movements and its long-run trajectory. That is why ee leave aside such macroeconomic determinants as fiscal and current account deficits.

We identify five main determinants: terms of trade (*TOT*), tradable to non-tradable price differentials (*TNT*, also known as the *Balassa-Samuelson effect*, *BSE*), interest rates differentials (*ID*), forward exchange rate (*f*) and risk premium (*ρ*), proxied by *VIX* international volatility that reflects the reward investors ask to hold a risky currency. While the former three have been extensively tested in empirical works (Clark and MacDonald [11], Ibarra [12], López Villavicencio and Raymond [13]), *f* and *ρ* have been left out of the picture. The joint consideration of these five factors is not common in the literature.

We argue that, while in the long run PPP theory tends to explain the path of the exchange rate, in view of the growing commercial and financial integration of the Mexican economy, financial variables have played an increasingly crucial role in determining movements in the nominal and real side of the currency. Foreign exchange markets, which operate with extremely high liquidity on a 24-hour basis and include spot and financial derivatives transactions, have gained immense importance as they have become the backbone of international financial wealth. For some years now, the Mexican peso has been used as a hedging currency for risks in currencies associated with lower liquidity or limited trading hours. According to BIS [14], the Mexican peso is the third most traded currency among emerging economies after the renminbi and the rupee. Its average daily trading volume during 2022 was US$114 billion dollars, well above US$1.6, US$0.16 and US$0.088 billion in exports, remittances and foreign direct investment, respectively.

In addition, it should be noted that 82% of transactions with Mexican pesos were carried out outside Mexico, 37% were in cash, and the rest (67%) were in financial derivatives, BIS [14]. All of this clearly indicates that the determination of the exchange rate in recent years has been the result of the appetite for the Mexican peso among the participants of the global foreign exchange market. On the other hand, we cannot underestimate the importance of the US monetary policy, which has generated massive and multiple financial and real effects around the world in recent decades.

Despite this major importance in the operation of foreign exchange markets, our econometric results show a significant, although still marginal, role of these variables.

While inflation targeting measures have been successful in maintaining price stability–compared to historical high inflation experienced in the 80’s–the floating exchange rate has increasingly been influenced by adjustments in international portfolios, especially in the aftermath of the *Great Financial Crisis* (GFC, 2008–2009).

In the early 2000s, the US federal funds rate decreased drastically (it plunged from 6.3% in 2000 to 1.1% in 2003), and both liquidity and sudden capital movements increased rapidly worldwide. Just after the bankruptcy of Lehman Brothers (September 15, 2008), with the first round of the so-called *Quantitative Easing* (*QE*) in November 2008, the Federal Reserve initiated a massive financial assets purchase program seeking to stabilize the economy and financial markets. The main purpose was to avoid at all cost a deflation like that in 1929–1932.

By the end of 2009, many other central banks adopted similar policies and the global macro-financial environment registered unprecedented levels of liquidity. As a result, emerging countries’ assets–such as the case of Mexico–became a target for the allocation of extra liquidity. According to Banco de México and INEGI data, non-residents holding government domestic securities amounted roughly to 1% of GDP in 2007 and this figure further jumped to 9% by the end of 2021 [15, 16]. Extraordinary monetary policies came to an end by 2012 and *hawkish* policies emerged and were followed by two years of adjustments in raw material prices, especially oil prices (West Texas Intermediate), which dropped from US$105 per barrel in June 2014 to US$30 in February 2016. The fall in oil prices did not attain the pre-2014 levels until March 2022 (US$108 per barrel [17]).

Our research begins in 2001.01, when the inflation targeting regime was adopted in Mexico, [18]. This allows us to capture an important phase of inflation reduction and control, and, therefore, a phase of homogeneous monetary policy. In this sense, while between 1995 and 2000 average inflation was 22.54% (with a standard deviation of 15.77) and the reference interest rate was 26.84% (with a standard deviation of 11.87), between 2001 and 2021 these figures attained 4.13% (1.18) and 6.03% (2.13), respectively.

Due to the increasing importance of international finance, we prove that *q* responds not only to the usual set of commercial fundamentals, but also to the unprecedented financial logic–i.e. expectations (proxied by *f*) and financial volatility (*ρ*, risk premium), proxied by VIX, which correspond to rational expectations, where *f* predicts the future movement in the nominal exchange rate MXN/USD. This theory has yielded mixed results in empirical studies [19]. According to Bush and López Noria [20], the currency risk premium for Mexico is strongly affected by international volatility.

Since we face a different order of integration among the five determinants of *q*, our ARDL Pesaran & Shin [21, 22] estimation finds a cointegrating vector only for 2009.01–2022.12, corresponding to *QE* as well as abundant international liquidity. Prior to 2009 and for the whole sample (2001.01–2022.12), our combination of commercial and financial determinants of *q* does not cointegrate. According to our econometric results, we suggest that *TNT* is the most important determinant of *q* in both the short and long run (-0.27 and -1.11, respectively), followed by *TOT* (-0.26 and -0.51). Besides, we find that our two financial determinants can alter *q* not only in the short run (as one would expect), but also in the long run, but sorpresilly in a marginal manner (0.0155 and 0.063 for *f*, and 0.009 and 0.036 for *ρ*, respectively). We also find high persistence effects of *q* on itself (0.75) over the four previous quarters.

In summary, using a combination of trade and financial variables, finding cointegration only for the period 2009.01–2022.12 and giving each factor its relative importance in the short and long terms are the main contributions of this work.

Section two reviews literature. Section three discusses theoretical issues of exchange rate determination. Section four depicts stylized facts (materials and methods) of the determinants of the MX peso–US dollar for 2001.01–2022.12. Section five addresses the econometric issues. Section six punctually presents the main results, and section seven concludes and gives further comments.

## Literature review

The vast majority of literature addresses the determinants of the real exchange rate from the perspective of macroeconomic, trade or financial aggregates, without mixing them; so, we have decided to take an eclectic approach and make a joint analysis of the importance of the latter two for Mexico in the period 2001.01–2022.12.

Traditionally, macroeconomic fundamentals were considered to be the sole (main) determinants of *q*. The examples of models that test them are Clark & MacDonald’s [11] Behavioral Equilibrium Exchange Rate (*BEER*) model, Stein’s [23] Natural Real Exchange Rate (*NATREX*), and Williamson’s [24] Fundamental Equilibrium Exchange Rates (*FEER*).

For a detailed theoretical explanation of these models, we highly recommend MacDonald, [25].

From the theoretical standpoint, the family of models that rely on macroeconomic fundamentals accepts the concept of exchange rate equilibrium as the level that equilibrates the balance of payments. However, they accept that in the short run there may be some degree of misalignment (volatility) of the nominal exchange rate (*E*) due to shocks in the macroeconomic fundamentals and financial shocks. In this sense Alagidede & Ibrahim [26] argue that for small economies characterized by institutional failures (such as Ghana and Sub-Saharan Africa) but with a possibility to extend to emerging economies in the short run, about three-quarters of real exchange rate shocks are attributed to the real exchange rate itself, and the rest, to the factors such as government spending, money supply, foreign direct investment flows, terms of trade and output shocks. Market failures can occur even in large emerging economies such as China. Chen *et al*. [27] argue that uncertainty regarding economic policy (associated with government regulation and decisions) is a definitive factor in explaining exchange rate volatility.

In the long run, however, they claim the equilibrium should be restored through macroeconomic (inner) adjustments and policies. According to this family of models, in the long run macroeconomic fundamentals drive *E*–and, consequently, *q*–towards the equilibrium and the effect of financial elements runs only in the short term.

As we have previously stated, in this article we leave the above approach aside and focus exclusively on trade and financial aspects, which have become increasingly important, particularly in emerging countries.

The exchange rate affects commodity prices and the welfare of the poorest share of the population in emerging economies where exchange rate volatility is higher than in developed countries, Nor *et al*. [28]. Yépez & Dzikpe [29] state that in emerging countries the relative prices of tradable goods account for most of the volatility observed in the exchange rate and that world commodity prices explain around 30% of their fluctuations.

For 1980–2014 and using a sample of 17 Latin American countries, Ramírez [30] suggests that increases in productivity cause an appreciation of the real exchange rate. However, the pass-through is not complete since a 1.0% increase in productivity causes an appreciation of 0.73% in *q*.

Catalán [31] uses an ARDL model applied to Mexico to confirm that the *Balassa-Samuelson Effect* is validated for 1994.I-2018.IV, which in author’s terms rejects the monetary model.

Kassouri and Altıntaş [32] argue that in Africa the exchange rate response to terms of trade’ shocks is asymmetric, because in the long run the response is stronger to positive shocks than to the negative ones.

In contrast, the portfolio approach literature argues that exchange rate fluctuations are determined by the portfolio rebalancing which, in turn, is influenced by risk premium, uncovered interest parity and investment decisions, Aliber [33] and Dooley and Isard [34]. In the same vein, Brainard and Tobin [35], Branson [36], Tobin [37] and Backus *et al*. [38] consider the exchange rate from the point of view of assets portfolio optimization, the so-called *portfolio approach*. This approach considers that financial assets differ in their country-specific risk premiums, so they are not perfectly substitutable. Then, *E* is determined by the desire to diversify portfolio according to risk preferences and expectations. In other words, the appetite of financial asset holders will be crucial to the supply and demand of currencies and, therefore, to the value of *E* in the short term.

In the opposite vein, Bostan *et al*. [39] argue that, at least in the case of Romania, the exchange rate serves as a reference for financial markets because it reflects competitiveness and its evolution is fundamental since it is influenced by an array of factors and, at the same time, its disruptions affect external competitiveness, the real economy and financial markets.

With respect to financial determinants, Cardozo *et al*. [40] mention for the case of Colombia the importance of the forward exchange rate and, by estimating EGARCH and VARX-MGARCH models, they find evidence that changes in the real sector impact the forward exchange rate. However, that effect has not been stable between 2008 and 2015.

Cáceres [41] states that the Mexican peso and the Peruvian Sol are risky currencies and are commonly used to do carry trade, a financial strategy that consists in financing with currencies associated with low interest rates in order to invest in high yield currencies associated with the risk premium. This type of strategies can lead to abrupt depreciations. Capasso *et al*. [42] argue that there are important links between the exchange rate and monetary policy.

### Theoretical issues

Uncovered interest parity (*UIP*) stands out as the pillar of the portfolio approach literature, which argues that expected exchange rate fluctuations are explained by interest rate differentials. The limited empirical evidence for the fulfillment of this theory led economists to develop the covered interest rate parity (*CIP*) as a possible explanation for exchange rate fluctuations. According to *CIP*, there is a close relationship between interest rates, spot rates and forward rates. Since assets are not homogeneous, investors hedge the currency risk through derivatives’ markets. If capitals are free to move, the *CIP* is an equilibrium condition that expresses the future exchange rate as the spot exchange rate adjusts for interest rates differentials. The information available in the forward market (*f*) allows players to account for the risk premium (*ρ*), typically originated by imperfect substitutivity of financial assets, while credit and bond markets include information about monetary policies across countries.

We follow Blecker’s [43] decomposition of the *CIP* rule from which it is possible to express the domestic interest rate on the international interest rate (i*), the expected depreciation of the local currency (Δ*e*^*e*^) and the risk premium (*ρ*):

$$i=i^{*}+\Delta e^{e}+\rho$$ (1)

We can further express the expected depreciation as:

$$\Delta e^{e}={(E}^{e}-E)/E$$ (2)

Where *E* and *E*^*e*^ are the nominal and the expected exchange rates, respectively. Plugging (2) into (1) and solving for *E*, we obtain:

$$E=E^{e}{\left[\left(i-i^{*}\right)+\left(1-\rho \right)\right]}^{-1}$$ (3)

In the short run, market participants adjust their portfolios according to arbitrage among markets. Covered interest arbitrage is carried out with operations between two currencies in the forward, spot and interest rate markets [44]. If this condition is met, the exchange rate benefits from a certain degree of stability, since the demand for currency would be conditioned by international trade, without the presence of speculative financial flows.

The condition of non-arbitrage, however, may not be fulfilled with the presence of transaction costs. In this case, three elements from (3) emerge: monetary policy (domestic and foreign), Δ*E*^*e*^, and *ρ*.

The monetary policy (domestic/international), measured as interest differentials, impacts portfolio flows. If the domestic rate (*i*) rises relatively to the international rate (i*), it will attract capital inflows, thus, appreciating the currency. However, this relationship may not always be fulfilled, since in the short run the reverse logic may apply as the interest rate could follow the movements of the exchange rate, and not vice versa. This occurs because the central bank, to tackle further nominal depreciation–and its pass-through effect on domestic prices–responds by raising interest rates. This phenomenon is known as the "fear of floating" [45].

Different expectations (Δ*e*^*e*^) might lead to speculative behaviors (changes in the appetite) and influence the demand for currency, [46]. For example, if traders believe the current value of *E* is not fully pricing some important factor, they might take speculative positions against it. This will lead to a consequent reallocation of financial flows that would alter *E* and *q*. This phenomenon has been called *self-fulfilling prophecies*, [43]. During bullish market phases, agents might commonly believe the market continues to expand, although the fundamental analysis may indicate different conclusions. During periods of excessive confidence or when the appetite for currency increases due to improved hedging, traders could buy domestic currency, causing a sustained appreciation and strong misalignment in macroeconomic fundamentals. As soon as agents become aware of the excess of confidence in the market, they rapidly dry out resources provoking depreciation. In finance, a common measure of currency expectations is the forward exchange rate (*f*) that consists in today’s price for future value for 3, 6, and 12-month horizons.

The forward rate is based on the difference between the interest rate of two countries and the time until the maturity of the deal. Forward points are calculated and transactions are executed for any date. They compensate for the difference in interest rates. For example, if the euro interest rate is 1% and the US interest rate is 2%, you could make the 1% difference by holding US dollars instead of euros [44].

By using (3) in the definition of *q*(EP*/*P*), it follows:

$$q=E^{e}{\left[\left(i-i^{*}\right)+\left(1-\rho \right)\right]}^{-1}\left(\frac{P^{*}}{P}\right)$$ (4)

Introducing two more equations for relative prices between countries (*P*, P*), as proposed by Barbosa *et al*. [45]:

$$P=P_{N}^{\alpha }P_{T}^{1-\alpha }={\left(\frac{P_{N}}{P_{T}}\right)}^{\alpha }P_{T}$$ (5)

$$P^{*}=P_{N}^{*\beta }P_{t}^{*1-\beta }={\left(\frac{P_{N}^{*}}{P_{T}^{*}}\right)}^{\beta }P_{T}^{*}$$ (6)

Where *P* and P* are aggregate prices in the domestic and foreign economy; *P*_*T*_, *P*_*N*_, $P_{T}^{*}$ and $P_{N}^{*}$ denote prices for tradable and non-tradable goods in the domestic and in the foreign country, and *α* and *β* are their weights. By replacing relative prices in *q*, we obtain:

$$q=E^{e}{\left[\left(i-i^{*}\right)+\left(1-\rho \right)\right]}^{-1}\left(\frac{P_{T}^{*}}{P_{T}}\right)\left[{\left(\frac{P_{N}^{*}}{P_{T}^{*}}\right)}^{\beta }/{\left(\frac{P_{N}}{P_{T}}\right)}^{\alpha }\right]$$ (7)

Eq (7) adds prices of tradable goods between the domestic and foreign country $\left(P_{T}^{*}/P_{T}\right)$ and the relationship between non-tradable sectors in the two economies $\left[{\left(\frac{P_{N}^{*}}{P_{T}^{*}}\right)}^{\beta }/{\left(\frac{P_{N}}{P_{T}}\right)}^{\alpha }\right]$.

By assuming that import prices of a country correspond to export prices of its commercial partner–which, given the trade interconnectedness between the United States and Mexico (82% of total Mexican trade is with the US), seems to be reasonable–then $\left(\frac{P_{T}^{*}}{P_{T}}\right)$ can be considered as the terms of trade (*TOT*), an important determinant of *q*. Improvements in export prices relative to import prices generate higher income from abroad, boosting consumption and, consequently, domestic non-tradable prices. Thus, in response to an improvement of *TOT*, there would be a decrease in *q* (appreciation). The negative relationship between *TOT* and *q* could also derive from capital flows. That is, when export prices grow and the economy is expanding, it may attract foreign capitals, which would appreciate *q* [46].

Additionally, Eq (7) includes relative price differentials of non-tradable goods between countries $\left[{\left(\frac{P_{N}^{*}}{P_{T}^{*}}\right)}^{\beta }/{\left(\frac{P_{N}}{P_{T}}\right)}^{\alpha }\right]$, known as the *Balassa-Samuelson Effect* (*BSE*) [47, 48], which considers that price differentials between the two sectors reflect productivity differences and swings in *q*. In addition, it argues that, if a country experiences permanent increases in productivity, then its currency will diverge from the PPP theory [49, 50]. That is, with higher productivity, non-tradable prices will be higher, and the currency will appreciate in real terms, shifting away from the law-of-one price trajectory.

All in all, and expressing *q* in reduced form in logs:

q=l+(f,-id,+ρ,-tot,-tnt) (8)

which denotes that *q* is a function (*l*) of: a) currency expectations (*f*), summarized by the forward exchange rate; b) the central banks’ 12-month nominal interest rates for US and Mexico differentials (*id*); c) the country risk premium (*ρ*); d) the terms of trade (*tot*); e) the *BSE* (*tnt*), that represents the ratio of relative prices of tradable to non-tradable goods in both countries. Following MacDonald [51], to capture the *BSE*, we construct the ratio of tradable to non-tradable prices. It is a composite index calculated as the relationship between the Consumer Price Index (*CPI*) and the Producer Price Index (*PPI*) for the domestic country (Mexico) and the foreign country (the United States). The *CPI* measures non-tradable prices and mainly refers to non-traded services and other goods. Conversely, the *PPI* approximates tradable goods used by the industrial sector. The variable is constructed as follows:

$$TNT=\frac{{CPI}_{t}∕{PPI}_{t}}{{CPI}_{t}^{*}∕{PPI}_{t}^{*}}$$

The asterisk refers to the United States’ variables. An increase in the index indicates that prices of non-tradable goods in Mexico are higher than those in the United States, appreciating *q*.

Kakkar [52] finds that, for 1955–1996, the bilateral real US-Mexico exchange rate is co-integrated with the relative Mexican and US prices of non-tradable goods. Therefore, changes in the inflation of non-tradable goods may explain permanent changes in *q*.

López Villavicencio and Raymond [13] adopt the macroeconomic fundamentals’ approach proposed by Clark and MacDonald [11] and test if productivity differential (*BSE*), current account, and interest rate differential with the US explain short- and long-run fluctuations in bilateral *q*. By estimating an ARDL model for 1960–2005, they provide evidence that improvements in productivity and increases in interest rate differentials appreciate the Mexican currency in the long run.

By using a Behavioral Equilibrium Exchange Rate model based on Clark and MacDonald [11] and MacDonald [53], Ibarra [12] develops an analytical model starting from the interest parity condition and tests it (1990–2006). In his work, cointegration analysis is used to test two determinants of *q* (prices and interest differentials with the United States), while controlling for the ratio of government consumption to GDP, manufacturing production ratio (as proxy of *BSE*) and oil prices. Using different specifications, the author shows that *q* depends negatively on the interest rate differential and positively on price differentials.

Loría *et al*. [54] test the monetary approach for the determination of *E*. By using a SVAR (1994–2007), they find that differentials between Mexico and the United States in output, inflation, and interest rate cause short- and long-run movements in *E*.

By using a VECM, López and Ventosa-Santaulària [55] prove the validity of *BSE*, and argue that the long-run depreciating trend of the Mexican peso (1980–2017) is attributable to the loss in productivity of the country relative to the United States.

### Materials and methods (stylized facts)

In order to find crucial regularities to be econometrically tested in the next section, in this section we briefly analyze the monthly evolution of the five main determinants of *q* here considered (January 2001 to December 2022).

**Fig 1:**
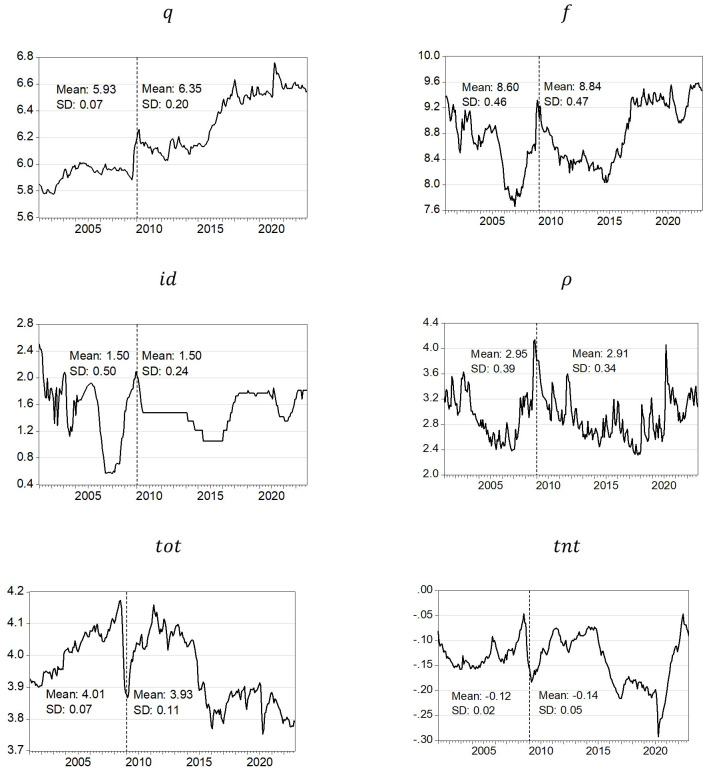
Determinants of *q*, 2001M01-2022M12. Note: own calculation, based on Banco de México [15], Bank for International Settlements [56], Bloomberg [44], BIS [14], FRED [17] and INEGI [16]. These sources are used for econometric estimates.

Fig 1 reports the evolution of *q*–calculated by Banco de México [15]–and its determinants. An increase in *q* represents depreciation and the opposite applies. According to our main hypothesis, the *GFC* marked two different paths, reason why we split the whole sample in two: 2001.01–2008.12 and 2009.01–2022.12. Ups and downs are more frequent and respond to major financial events. The normalization of monetary policy in the US, the outcome of the US presidential elections (*Trump effect*) as well as commercial tensions and NAFTA renegotiations provoked further depreciation and volatility (2014–2017). It is striking that, if we eliminate the strong Covid-related depreciation in 2020, the Mexican economy has exhibited a clear trajectory of exchange rate appreciation (until April 2023) that is definitely explained by the increase in *id* despite the strong deterioration of *tot* and the advance of *tnt*. Said phenomenon of a strong appreciation over the past three years would support our main hypothesis.

The variables show a unit root behavior with the exception of *id* and *ρ* that behave as I(0), Table A1 in S1 Appendix. That is why the ARDL [21, 22] procedure is convenient.

**Fig 2:**
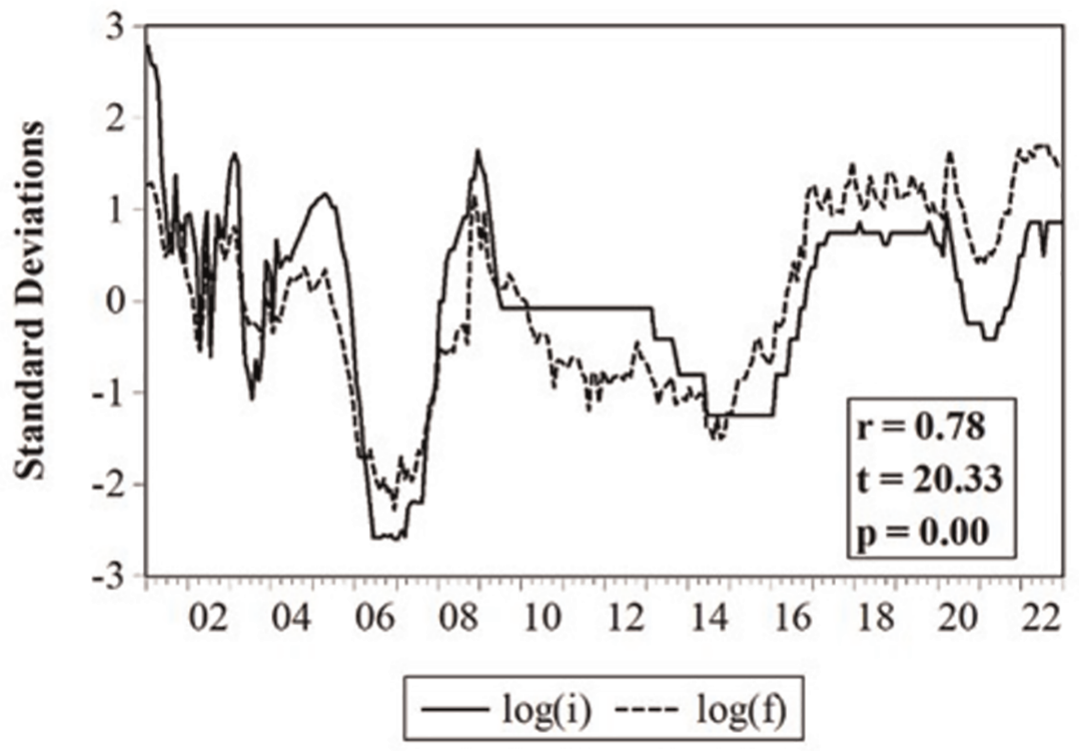
Interest rate differentials and one-year forward rate, 2001.01–2022.12. Note: In order to contrast visually, both series were normalized by their means and standard deviations.

Fig 2 clearly illustrates the strong relationship between *id* and *ρ*, which confirms the covered interest parity theory in explaining currency fluctuations, and the need to state a correct specification by eliminating *id* in the econometric estimation.

### Econometric issues

ARDL models [21, 22] are linear time series models in which both dependent and independent variables are related historically and contemporaneously. These models have gained popularity in recent years as a method of examining robust cointegrating relationships between variables of different orders of integration. They are highly informative since they offer: a) an intertemporal dynamic estimation (given that they are also closely related to Vector Autoregressions), b) derivation of the long-run (equilibrium) relationship, and c) the conditional error correction.

Since we have a different order of integration and to avoid spurious regressions, by applying the *Bound F-test*, we can claim that the whole set of variables is cointegrated only for 2009.01–2022.12, Table 1.

**Table 1:** Cointegration (Bound F-test) tests, 2001.12–2022.12.

Period	F-Statistic	Cointegration Ho	ARDL Selection
2001.01–2022.12	3.64*	∄	(3,3,3,1,2)
2001.01–2008.12	2.31**	∄	(1,3,3,1,4)
2009.01–2022.12	7.53***	∃	(3,3,0,0,2)

H_0_: No levels relationship.

* Not valid at 2.5%.

** Not valid at 10%.

***Valid at 1%. We followed the AIC.

According to our approach, if *y*_*t*_ is the dependent variable (*q*) and *x*_*j*,*t*−*z*_ are explanatory variables, a general ARDL model can be represented as follows:

$$y_{t}=a_{0}+a_{1}t+\sum_{i=1}^{p}\varphi_{i}y_{t-i}+\sum_{j=1}^{k}\sum_{z=0}^{q_{z}}\beta_{j,z}x_{j,t-z}+\epsilon_{t}$$ (9)

Where *a*_0_, *a*_1_, *φ*_*i*_, and *β*_*j*,*z*_ are the coefficients associated to the constant, to a linear trend, to the lags of *y*_*t*_ and to the lags of regressors *x*_*j*,*t*−*z*_, respectively for *j* = 1, …, *k*; *ϵ*_*t*_ are Gaussian innovations.

The model that minimizes the AIC is the ARDL (4,2,2,0,0,1,0). Tables 2 and 3 report short-run estimates. Since all variables are expressed in their logarithms, they evaluate constant elasticities.

**Table 2:** Short-run deteminants of *q*, 2009.01–2022.12.

Variable	coef	t-stat	Prob
c	1.75	6.38	0.00
*q(-1)*	1.021	16.88	0.00
*q(-2)*	-0.330	-4.93	0.00
*q(-3)*	0.112	2.52	0.00
*q(-4)*	-0.051	-1.85	0.06
*tot*	-1.003	-17.78	0.00
*tot(-1)*	1.211	10.91	0.00
*tot (-2)*	-0.334	-3.94	0.00
*tnt*	-0.695	-6.24	0.00
*tnt(-1)*	0.421	3.49	0.00
***ρ***	0.009	3.54	0.00
*f*	0.060	5.76	0.00
*f(-1)*	-0.045	-4.41	0.00
*t*	0.0005	5.45	0.00
*D*	-0.0341	-8.59	0.00

*R*^2^ = 0.998; DW = 1.98; LM(4) = 0.2557(0.9058); JB = 0.2195(0.8961); Breusch-Pagan-Godfrey = 0.9615(0.4953); RESET(2) = 2.6024(0.0774). According to CUSUM, CUSUMQ and Recursive Residuals, there is no evidence of a structural break (CUSUMQ). D = Dummy variable to achieve correct specification without economic meaning. All estimations were made with EViews 13.

**Table 3:** Overall short-run estimates.

*Variable*	Coef
*q*	0.7525
*tot*	-0.1264
*tnt*	-0.2742
***ρ***	0.0090
*f*	0.0155
*t*	0.0005
*D*	-0.0340

## Results

As expected, when both *f* and *id* are included, strong collinearity problems emerge, which caused *id*, *q*_*t*-3_, *q*_*t*-4_ and *tnt*_*t*-2_ to become non-significant, hence, we eliminate *id*. The estimation confirms that there are important autoregressive processes that in all cases are significant at 1%, with the exception of *q*_*t*-4_, which is significant at 5%. In addition, we test for omitted variable (*H*_0_: *id* = 0), which cannot be rejected (*F stat* = 0.7118(0.4002)), and prove that *f*_*t*_ and *f*_*t*-1_ are jointly redundant (*H*_0_: *f* = 0; *F stat* = 20.55(0.0000)), which is clearly rejected, all of which reinforces our specification.

After eliminating non-significant regressors, we find that the constant and the linear trend (*t*) confirm the long-run depreciation trajectory over time.

According to all the correct specification statistics, our model fits the data well so we can fairly claim that it is a good approximation to Data Generating Process, Hendry [57]. To have a better picture of the above reported results, we summarize the overall short-run effects by summing up all coefficients for each variable and report them in Table 3. The strong (dynamic) persistence effect of *q* stands out in its own determination, which is above that of the other variables. Next in importance are *tnt* and *tot* and, finally, significant–albeit marginal–effects of *ρ* and *f* are notable.

Table 4 reports the long-term equilibrium relation, since the cointegration error coefficient (*ECM*) is correct and significant and indicates that 25% of a shock occurred today is absorbed within the next month in the cointegrating relationship.

**Table 4:** Long-run estimates.

Variable	Coef	t-stat	Prob
*ECM*	-0.2475	-11.41	0.00
*tot*	-0.5106	-8.45	0.00
*tnt*	-1.1080	-13.53	0.00
***ρ***	0.0361	3.17	0.00
*f*	0.0626	5.02	0.00
*t*	0.0020	14.96	0.00
*D*	-0.1379	-5.62	0.00

In general terms, estimates in Table 4 confirm the short-run outcome at 1% in all cases. The same order of importance of the determinants of *q* is observed, and short-term proportions are maintained, although it stands out that all parameters are much higher, and the elasticity of *tnt* is now above unity.

Our approach and results are largely consistent with Catalán [31] who sustains that exchange rate fluctuations cannot be explained in a conventional manner through the supply and demand approach, which would be applied to any good, because economic agents’ perception of the value of each currency is highly relevant.

Similarly, our results, in part, coincide with the PPP approach [31], which we verified based on the depreciation trend of the real exchange rate with the coefficient of 0.0020.

Finding a period of cointegration is very important because it is not a general condition, as in the case of Canada [31]. The importance of this result lies in that this evidence supports the presence of the *Balassa-Samuelson Effect* in the long run, which implies the rejection of the monetary model [31] mainly because of the relationship between tradable and non-tradable goods. Yépez & Dzikpe [29] testify to this feature by mentioning that in emerging countries the relative prices of tradable goods account for most of the volatility observed in the exchange rate, which is approximately 30%.

With respect to *tot*, which we place in the second place of relevance, Ramírez [30] shows that increases in productivity lead to an appreciation of the real exchange rate with an elasticity of 0.73.

Finally, we mention the effect of financial variables (which we recover as the *forward exchange rate* and *the risk premium*). Cardozo *et al*. [40] analyze the forward exchange rate for Colombia and conclude that, despite being an important variable in explaining the exchange rate, its relevance declined in the 2008–2015 period. This stands in contrast to Cáceres [41] who points out that the Colombian peso lacks the importance that the Peruvian Sol and the Mexican peso have in international markets to apply carry trade strategies. Therefore, these financial variables are more relevant for the case of Mexico. The latter result reinforces the position of the Mexican Peso as the third most traded currency among emerging markets [14].

## Concluding remarks and further comments

We study and estimate short- and long-run determinants of the real bilateral (MEX-US) exchange rate (*q)* for the Mexican economy (2001.01–2022.12). We define five variables (in logs) as their main drivers: terms of trade (*tot*), price differentials (*tnt*), interest rate differentials (*id*), forward exchange rate (*f*) and risk premium (*ρ*).

Given the different order of integration, an ARDL model (4,2,2,0,0,1,0) was estimated, which confirms cointegration and short- and long-run effects only for 2009.01–2022.12.

We estimate short–and long-run interactions. In both cases, the *Balassa-Samuelson Effect* (*tnt)* turned out to be the main determinant of *q* (-0.27 and -1-11 for the short and long run), followed by *tot* (-0.126 and -0.51), *f* (0.005 and 0.063), and *ρ* (0.009 and 0.030), respectively. All in all, an increase in domestic relative prices, as well as an improvement in terms of trade, lead to *q* appreciation, while increases in *f* and *ρ* generate depreciation. Due to high collinearity, interest rate differentials (*id*) are not statistically significant and were, thus, excluded from estimations, following a detailed econometric procedure.

The results corroborate our hypothesis that, in the aftermath of the *Great Financial Crisis* and coincidently with the abundance of international liquidity, the depreciation/appreciation trajectory of *q* has been gradually influenced by financial determinants.

The current international environment is still characterized by an unprecedented level of liquidity, generated in response to the *coronacrisis*. Therefore, investors around the globe continue reallocating “cheap money” basically to emerging markets. In this continuously expanding financial environment, currency volatility in all countries is to be expected as a norm, rather than a signal of unusual events. Indeed, not only asset managers have disposable money, but also government, private firms and households’ debt issuance have been growing exponentially. The research of short-run gains of financial investors jointly with the possible mismanagement of debt (both public and private) could generate dangerous dynamics, which often lead to a well-known result: the currency crisis, which strongly affects real variables, which, in turn, depress the living standards in the long run.

Although financial determinants are second in importance for the whole estimation period, they are very likely to continue gaining relative weight in the current context of high liquidity and uncertainty and high appreciation of the Mexican peso. This seems to be related to the increased appetite for the Mexican Peso over the last three years, which is clearly associated with the increase in the forward rate and the interest rate differential.

Although the determinants of the long-term exchange rate respond to PPP, in the short-term financial instruments have a significant impact on it, so it is highly probable that as long as Mexico’s reference rate remains 6 points higher than the US rate (as it has occurred until April 2023), it is expected to ensure a relatively stable nominal and real exchange rate and to continue appreciating with a downward effect on inflation.

## Supporting information

S1 Appendix (DOCX)
