# Supplementary material for: Mexico: Determinants of the real exchange rate, 2001.01–2022.12
Source: PLoS One. 2023 Dec 6;18(12):e0286331. doi: 10.1371/journal.pone.0286331 (PMC10699617; doi:10.1371/journal.pone.0286331)
Supplement: S1 Appendix — (DOCX) [file pone.0286331.s001.docx]

**Appendix**

|  | *log(q)* | | *log(f)* | | *log(id)* | | *log(*$\rho$*)* | | *log(tot)* | | *log(tnt)* | |
| --- | --- | --- | --- | --- | --- | --- | --- | --- | --- | --- | --- | --- |
|  | Level | $\Delta$ | Level | $\Delta$ | Level | $\Delta$ | Level | $\Delta$ | Level | $\Delta$ | Level | $\Delta$ |
|  | ADF | | | | | | | | | | | |
| Trend & Intercept | -2.95 (0.15) | -12.17 (0.00) | -2.20 (0.49) | -16.22 (0.00) | -4.28 (0.00) | -13.71 (0.00) | -4.21 (0.01) | -10.86 (0.00) | -3.44 (0.05) | -9.29 (0.00) | -1.41 (0.86) | -9.11 (0.00) |
| Intercept | -0.87 (0.80) | -12.19 (0.00) | -1.67 (0.45) | -16.15 (0.00) | -4.27 (0.00) | -13.74 (0.00) | -4.3 (0.00) | -13.38 (0.00) | -1.38 (0.59) | -9.23 (0.00) | -1.44 (0.56) | -9.14 (0.00) |
| None | 1.47 (0.97) | -12.07 (0.00) | -0.03 (0.67) | -16.18 (0.00) | -0.53 (0.49) | -13.76 (0.00) | -0.84 (0.35) | -13.41 (0.00) | -0.39 (0.54) | -9.25 (0.00) | -0.85 (0.35) | -9.15 (0.00) |
|  | PP | | | | | | | | | | | |
| Trend & Intercept | -2.98 (0.14) | -12.07 (0.00) | -2.28 (0.45) | -16.21 (0.00) | -4.08 (0.01) | -19.90 (0.00) | -3.97 (0.01) | -18.61 (0.00) | -3.00 (0.13) | -9.00 (0.00) | -1.33 (0.88) | -9.15 (0.00) |
| Intercept | -0.89 (0.79) | -12.10 (0.00) | -1.80 (0.38) | -16.15 (0.00) | -4.05 (0.00) | -19.88 (0.00) | -4.1 0(0.00) | -16 (0.00) | -1.02 (0.74) | -8.98 (0.00) | -1.41 (0.57) | -9.17 (0.00) |
| None | 1.50 (0.97) | -12.07 (0.00) | -0.03 (0.67) | -16.18 (0.00) | -0.34 (0.56) | -19.94 (0.00) | -1.00 (0.28) | -15.97 (0.00) | -0.35 (0.56) | -9.01 (0.00) | -0.83 (0.36) | -9.18 (0.00) |

**Table A1. Unit root test, 2001.01-2022.12** Ho: ∃ unit root, probability in parentheses. Estimates in logs. For the correct specification of the ADF test, the Schwartz criterion was followed (14 lags max) and, for the PP test, the Bartlett Kernel criterion (3 lags max) was used. By the procedure of Dolado, Jenkinson and Sosvilla-Rivero. Enders [1], it is concluded that $q, f, tot$ and $tnt$ are I(1) in levels and stationary in their first differences, while $id$ and $\rho$ are stationary in levels. I thank Emmanuel Salas for conducting these tests.

1. Enders W. Applied Econometric Times Series. 4^th^ ed. Wiley; 2014.
